# Dynamics of Structural and Functional Changes in Gut Microbiota during Treatment with a Microalgal β-Glucan, Paramylon and the Impact on Gut Inflammation

**DOI:** 10.3390/nu12082193

**Published:** 2020-07-23

**Authors:** Harrison B. Taylor, Radhika Gudi, Robert Brown, Chenthamarakshan Vasu

**Affiliations:** Department of Microbiology and Immunology, College of Medicine, Medical University of South Carolina, Charleston, SC 29425, USA; taylorha@musc.edu (H.B.T.); gudi@musc.edu (R.G.); brownr27@winthrop.edu (R.B.)

**Keywords:** microbiota, paramylon, β-glucan, gut mucosa, gut inflammation, immune modulation, immune regulation

## Abstract

Previously, we have shown that oral administration of yeast derived β-1,3/1,6-d-glucan enhances immune regulation and alters the composition of the gut microbiota. However, it is not known if other structurally distinct β-glucans have similar properties. Here, using C57BL/6 mice, we show the potential of a microalgae derived β-1,3-d-glucan, paramylon (PM), in shaping the gut microbiota and modulating the susceptibility to colitis. The community structure within the gut microbiota showed progressive changes including selective enrichment of specific communities and lowered community richness and diversity during prolonged oral treatment with PM. Compared to control mice, the gut microbiota of PM-treated mice had significantly higher abundance of Verrucomicrobia and lower abundance of Firmicutes. Specific taxa that were significantly more abundant in PM-treated mice include *Akkermansia muciniphila* and several *Bacteroides* members. Predictive functional analysis revealed overrepresentation of carbohydrate metabolism function in the fecal microbiota of PM recipients compared to controls, and this function was linked to *Bacteroides* spp. Prolonged pretreatment with PM not only diminished susceptibility to dextran sulfate sodium induced colitis severity, but also caused enhanced immune regulation. Overall, this study demonstrates the prebiotic properties of PM and the potential benefits of its prolonged oral consumption to gut health.

## 1. Background

Prebiotic supplementation has long been used for promoting digestive health and regularity through manipulation of the gut microbiome [1,2]. One group of such compounds are β-glucans (BGs), which are complex polymers of D-glucose held together primarily via β-1,3 glycosidic linkages, with differing patterns of bonds such as β-1,4 and β-1,6 linkages or branching distinguishing the different types of BGs [3,4,5]. These non-digestible molecules can be extracted from many sources, including yeast [4,5,6,7], barley and oats [8,9], and algae [3,10]. Many health benefits of these complex dietary polysaccharides (CDP), including lowering of low-density lipoprotein (LDL) cholesterol [11,12], immune system stimulation and maintenance of immune homeostasis [3], attenuation and prevention of autoimmunity through increased anti-inflammatory cytokine production [4,5], anti-tumor activity [13,14], and short-chain fatty acid (SCFA) production [15] have been reported. Key immune system modulating health benefits observed with β-glucans, at least in part, appear to be through its direct binding to Dectin-1, a pathogen recognition receptor [3,16]. Our research on autoimmune diseases has additionally shown Dectin-1 or gut microbiota-dependent preventative and protective effects of systemic treatment with yeast β-glucan (YBG; β-1,3/1,6-d-glucan) in type 1 diabetes (T1D) [17] and oral treatment with YBG in T1D [4] and colitis [5].

Previous reports, including ours, have found that YBG and barley β-glucan (β-1,3/1,4-d-glucan) administration can significantly alter the gut microbiota in mouse models [4,5] and humans [8], respectively. In mouse models of T1D and colitis, oral treatment with YBG enriched Bacteroidetes and Verrucomicrobia and diminished the phylum Firmicutes [4,5]. At the genus level, *Akkermansia* spp. (Verrucomicrobia) and *Parabacteroides* spp. (Bacteroidetes) were found to increase significantly after treatment, while *Oscillospira* (Firmicutes) decreased after treatment. Functional predictions of the gut microbiota of yeast β-glucan-treated mice revealed an upregulation in functions linked to the utilization and metabolism of β-glucans after treatment. Treatment with oat and barley-derived β-glucans have been found to stimulate and increase the levels of probiotics like bifidobacteria and lactobacilli in the gut, with oat-derived β-glucan having a more profound influence compared to barley-derived β-glucan [18]. Similar shifts in Bacteroidetes to Firmicutes ratio were observed in human subjects who received high molecular weight β-glucan derived from barley [8]. Further, in silico predictions showed enhanced functions of microbiota related to glycan utilization and glycerolipid metabolism after treatment with these cereal derived β-glucans.

While yeast and cereal derived β-glucans have been studied in conjunction with their effects on the gut microbiota in relation to cardiovascular and immune health [4,5,8,19], not much is known about the effects of a widely used microalgae-derived β-glucan, paramylon (PM; β-1,3-d-glucan) on the gut microbiota and gut immune function. PM is produced by *Euglena gracilis* as a storage molecule that exists primarily as crystallized granules within the cell [20,21,22]. One distinguishing feature of PM compared to other β-glucans is its high yield, typically making up at least 90% of the dry weight of the host cell [22]; hence, it is considered easier to extract a highly purified version of β-glucan. In a study examining obesity in C57BL/6 mice, inclusion of paramylon into a high fat diet improved obesity outcomes via increased relative levels of *Bifidobacterium* and *Lactobacillus* [23]. A study examining the effect of laminarin or paramylon in Sprague-Dawley rats [24] concluded that differences in digestive system and gut microbiome responses to different β-glucans are likely due to differences in size, weight, and solubility.

In this study, we determined the progressive changes in microbiota structure and predictive function in C57BL/6 mice during treatment with highly purified (>99%) PM. We observed that prolonged oral treatment with PM significantly altered both the composition and function of the gut microbiota, with increases in *Bacteroides* and *Akkermansia* taxa and functions potentially linked to carbohydrate metabolism. Conversely, we found a decrease in the compositional levels and functions linked to members of Firmicutes after PM treatment. Importantly, pretreatment of mice with PM resulted in enhanced immune regulation and suppressed susceptibility to chemical induced colitis. This study, for the first time, demonstrates the prebiotic properties of a microalgae derived β-1,3-d-glucan and how its consumption may influence the gut environment both microbiologically and immunologically.

## 2. Methods

### 2.1. Mice and Treatments

C57BL/6 (B6) mice were originally purchased from the Jackson Laboratory (Bar Harbor, ME) and bred within the specific pathogen-free (SPF) facility at the Medical University of South Carolina (MUSC). Mice were maintained on a standard autoclaved rodent diet and autoclaved water. Euthanasia was performed by using CO_2_ with a flow rate about 20% displacement/minute. All studies were conducted under the protocol #3064 and this protocol was approved by the Ethics Committee (institutional animal care and use committee; IACUC). For microbiota analysis, female mice aged 8 weeks were administered either a 0.9% saline solution as control (*n* = 4) or 250 μg of PM (Millipore-Sigma; cat# 89862) in saline (*n* = 5) per day, for 45 consecutive days, via oral gavage. This dose, which is about 10 mg/kg body weight, was selected because it is comparable to the recommended human dosage of β-glucan dietary supplements (500–1000 mg per day). Our previous reports (4,5) have shown that this dosage of BG is effective in modulating gut immune function. Fecal pellets were collected from individually housed mice at day 0 (prior to treatment), 15 days, 30 days, and 45 days post-treatment and stored at −20 °C. Bodyweights of both groups mice were measured every 3rd day to determine changes in eating habits (Supplemental Figure S1). In some experiments, mice received treatment for 3 consecutive days.

### 2.2. Analysis of the Impact of PM Pretreatment

Cohorts of female B6 mice were each administered either saline (*n* = 5) or 250 μg PM (*n* = 5) suspension daily, via oral gavage, for 55 days. At day 45, mice received 2.5% (*w*/*v*) dextran sulfate sodium (DSS) to induce colitis until day 50, when they were switched back to regular water for the remainder of treatment. Mouse body weight was measured on day 45 (pre-treatment with DSS) and every day thereafter. At day 55, mice were euthanized and their colons harvested and their lengths measured. Sections of the distal colon were subjected to hematoxylin and eosin (H&E) staining and severity was evaluated as described previously [5]. Mesenteric lymph nodes (MLN) and colon tissues from saline (*n* = 5) and PM (*n* = 5) treated female mice were harvested for analysis of immune response to PM treatment. Fluorescence-activated cell sorting (FACS) analysis was performed on MLN cells that were stained for Foxp3 or MLN cells activated using PMA/Ionomycin with Brefeldin A for 4 h and stained for different cytokines. From the colon tissues, immune cells were enriched from single cell suspensions as described before [4,5] and cultured with anti-CD3 antibody for 24 h and then subjected to Luminex technology based multiplex assay to determine the concentration of cytokines. For qPCR assay, RNA preparations were made from distal ileum and colon tissues from cohorts of female mice that received saline (*n* = 5) or PM (*n* = 5) for 45 days using TRIZOL reagent (Invitrogen) and cDNA were synthesized using Superscript first-strand cDNA kit (Invitrogen). qPCR assay using factor specific primer sets were carried out as described in our recent reports [4].

### 2.3. 16S Ribosomal RNA Gene Sequencing and Bacterial Community Analysis

DNA was extracted from fecal pellets as described previously [4,5,25] and the V3-V4 region of the 16S ribosomal RNA (rRNA) gene was sequenced on an Illumina MiSeq platform in the genomic center at MUSC. Taxonomic classifications and diversity analysis were performed on the resulting sequencing reads using the web-based sequencing analysis software MicrobiomeAnalyst (McGill University, Quebec, Canada) with the Marker Data Profiling feature [26]. A BIOM file, metadata file, and phylogenetic tree file were uploaded, and data filtering was performed. Normalization of the sequences by rarefying to the minimum library size (5164 sequences) and total sum scaling was then performed to eliminate the disparity of unequal sequence depth. Taxonomic classification was performed against the GreenGenes database [27] and grouped into operational taxonomic units (OTUs) and classified based upon a 97% identity cutoff to reference sequences within the database. Sequencing data were also used as described previously [5] for the predictive functional analysis of the Kyoto encyclopedia of genes and genomes (KEGG) orthologs using PICRUSt [28,29], which assigned functions to three levels of functional categories, which were then analyzed using Statistical Analysis of Metagenomic Profiles (STAMP; Dalhousie University, Nova Scotia, Canada) software package [30]. Additional analysis of predicted functions was performed using the Global and Local Mapper features on the iVikodak platform [31].

### 2.4. Statistical Analysis

Statistical analyses including calculation and comparison of means, standard deviation (SD), and statistical significance (*p*-value) were performed and figures constructed using EXCEL (Microsoft, Redmond, WA, USA), STAMP [30], and GraphPad Prism (San Diego, CA, USA) applications. A *p*-value of ≤0.05 was considered statistically significant. Welch’s *t*-tests were implemented in GraphPad Prism to test the significance between treatments unless otherwise stated. *p*-values were corrected in STAMP using the Benjamini and Hochberg correction method [32]. The significance in α-diversity and β-diversity between samples was analyzed in MicrobiomeAnalyst via Mann–Whitney and permutational multivariate ANOVA (PERMANOVA) methods, respectively. Statistical significance was calculated via unpaired *t*-test for bodyweight at each time point. Fisher’s exact test was employed for comparing inflammation scores. A Mann–Whitney test was used for comparing data from immune cell assays. A false discovery rate (FDR) value of 0.05 was used while determining *p*-values.

## 3. Results

### 3.1. Prolonged Oral Administration of PM Alters the Structure of Gut Microbiota

**Figure 1:**
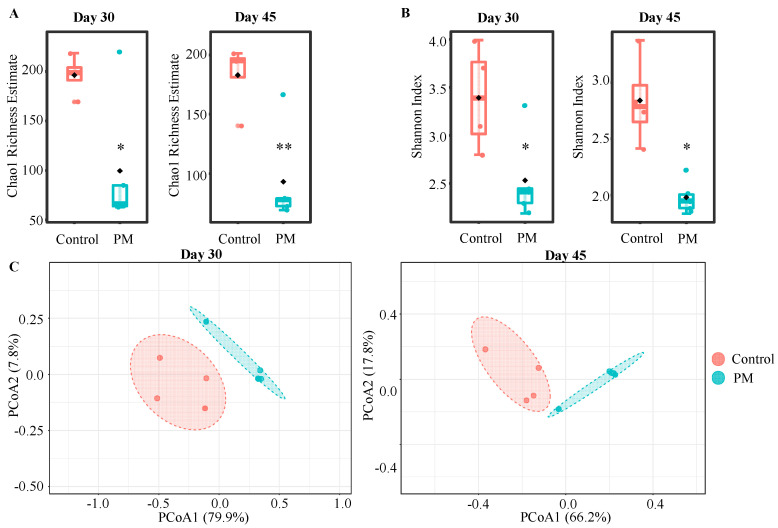
α and β diversity of the fecal microbiota in control and PM-treated mice. α and β diversity analyses were performed using 16S rRNA gene sequencing data of fecal samples from control (*n* = 4) and PM-treated (*n* = 5) mice collected before (day 0) and at various time-points (days 15, 30 and 45) during the treatment. Chao1 estimates and Shannon indices were employed to evaluate richness and diversity within each sample, respectively, as shown in Supplemental Figure S2. Differences in richness (**A**) and diversity (**B**) indices between treatments at day 30 and 45 are shown here. Statistical significance was assessed using *t*-tests. * *p* < 0.05 and ** *p* < 0.01 (**C**) Principal coordinate analysis (PCoA), by employing Bray–Curtis dissimilarity approach, examining the community structure differences between samples of control and PM treatment groups collected on days 30 and 45. Statistical significance was evaluated using PERMANOVA and mentioned in the text.

To determine the impact of consuming highly purified PM on gut microbiota, B6 mice were treated with this agent by oral gavage for 45 consecutive days. The pre- and post-treatment fecal samples from control and PM recipients were collected and examined for progressive changes in the profiles of microbial communities by 16S rRNA gene sequencing. An analysis of alpha (α) and beta (β) diversities revealed differences in the overall structure of gut microbiota of control and PM-treated mice. Although gut microbiota appeared to be unaltered initially (15d), between day 15 and day 30 of treatment, the average richness of the gut microbiota significantly decreased in both control (*p* < 0.001) and PM-treated mice (*p* < 0.001), based on Chao1 richness estimator (Supplemental Figure S2A). While this change appears to be, in part, due to age dependent maturation and/or unknown changes in the environment, the degree of decrease in richness was significantly greater in PM-treated mice compared to controls at both 30 (*p* = 0.0302) and 45 (*p* = 0.0064) days (Figure 1A). Further, Shannon index estimates (Supplemental Figure S2B) revealed a significant decrease in diversity at day 30 in both control (*p* = 0.0101) and PM-treated mice (*p* < 0.0001). In PM-treated mice, diversity decreased significantly further at day 45 (*p* = 0.034) from day 30. Diversity was significantly lower in PM-treated mice compared to controls at both 30 (*p* = 0.045) and 45 (*p* = 0.018) days (Figure 1B).

β diversity was measured to evaluate the overall differences in community structure between control and PM treatment groups via Principal Coordinate Analysis (PCoA) using the Bray–Curtis index with PERMANOVA. These analyses revealed spatially distant clustering of most of the day 30 and day 45 samples from control and PM-treated groups compared to pre-treatment and 15-day time-points (Supplemental Figure S2C). In PM-treated mice, compared to controls, samples from day 45 showed more spatial separation from day 30 samples. When samples grouped according to the duration of treatment were compared, significantly distinct clustering was observed between control and PM-treated mice at both the day 30 (*p* = 0.018) and day 45 (*p* = 0.013) time-points (Figure 1C). These observations suggest that while the gut microbiota in B6 mice used in this study appeared to have undergone maturation, PM treatment had a significant impact on its overall structure and composition.

### 3.2. PM Treatment Results in Changes in the Fecal Microbial Community Profile

**Figure 2:**
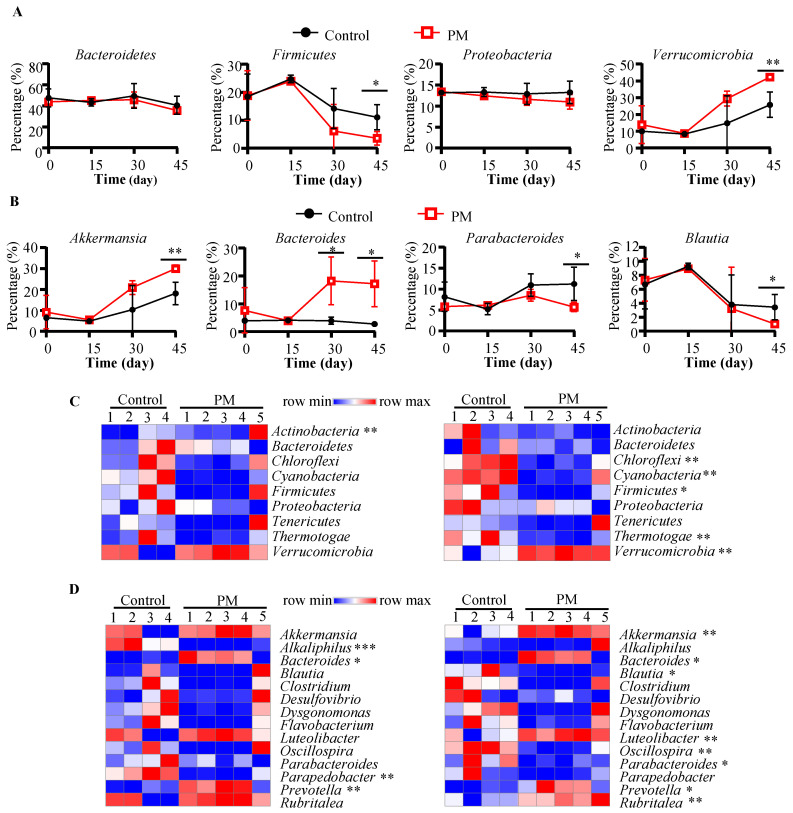
Time-dependent changes of fecal microbiota in control and PM-treated mice. Relative abundances of microbial communities at the Phylum and Genus levels were determined in fecal samples collected from control (*n* = 4) and PM-treated (*n* = 5) mice before (day 0) and various time points (days 15, 30, and 45) during the treatment. Analysis of time-dependent changes in major phyla (**A**), and genera (**B**) between control and PM-treated mice are shown. Relative abundances of microbial communities at the phylum (**C**) and genus (**D**) levels in fecal samples collected from control and PM-treated mice on day 30 (left panels) and day 45 (right panels) are also shown. Heat maps were generated using Morpheus (Broad Institute, Cambridge, USA), *p*-values by unpaired *t*-test. *p*-values were determined using unpaired *t*-test on GraphPad Prism. * *p* < 0.05, ** *p* < 0.01 and *** *p* < 0.001.

Next, we examined if 16S rRNA gene sequencing data of control and PM-treated mice show differences in the profiles of gut microbial communities. Compilation of OTUs to different taxonomical levels revealed distinct patterns in gut microbial composition between control and PM treatments only at day 30 and day 45 time-points. At the phylum level (Supplemental Figure S3A), the gut microbiota in general was primarily comprised of Bacteroidetes (31.6 to 64.7%), Firmicutes (1.5 to 26.7%), Proteobacteria (8.1 to 16.2%), and Verrucomicrobia (0.4 to 45.1%). While Verrucomicrobia was the only phylum that showed progressive increase in abundance between day 0 and day 45 (*p* = 0.0062 for control and *p* = 0.0005 for PM-treated), the increase on day 45 was significantly higher in PM-treated mice compared to controls (*p* = 0.002; Figure 2A). Compared to day 0, other key changes observed at day 45 in PM-treated mice include significant decreases in Bacteroidetes (*p* = 0.0004), Firmicutes (*p* = 0.0059), and Proteobacteria (*p* = 0.0179). No significant differences in these phyla were observed between days 0 and 45 in control mice. Importantly, the day 45 abundance of Firmicutes was significantly lower in PM-treated mice compared to their control counterparts (*p* = 0.0147).

At the genus level (Supplemental Figure S3B), the most abundant taxa represented in the gut included *Akkermansia*, *Bacteroides*, *Blautia*, and *Parabacteroides*. Changes in fecal microbiota over time include significant increases in *Akkermansia* (*p* = 0.0056 for control and *p* = 0.0005 for PM-treated mice), *Luteolibacter* (*p* = 0.005 for control and *p* = 0.002 for PM-treated mice), and *Rubritalea* (*p* = 0.009 for control and *p* = 0.00095 for PM-treated mice), and significant decreases in *Clostridium* (*p* = 0.0158 for control and *p* = 0.0186 for PM-treated mice), *Flavobacterium* (*p* = 0.006 for control and *p* = 0.008 for PM-treated mice), and *Oscillospira* (*p* = 0.026 for control and *p* = 0.006 for PM-treated mice) between days 0 and 45. Importantly, significant time-dependent changes observed only in PM-treated mice include decreases in *Blautia* spp. (*p* = 0.0018) and *Parapedobacter* (*p* = 0.0087). Significant differences also existed at day 45 between control and PM-treated mice at the genus level (Figure 2B). These included significantly higher levels of *Akkermansia* (*p* = 0.0024) and *Bacteroides* (*p* = 0.0106) and lower levels of *Parabacteroides* (*p* = 0.02) and *Blautia* (*p* = 0.02) in PM recipient mice compared to controls. Of note, comparison of microbial community profiles of day 0 and day 15 time points within and between groups showed not many differences, with the exception of an increase in *Parabacteroides* sp. and *Sutterella* sp. in the PM-treated mice at day 15 compared to pretreatment time-point (data not shown).

### 3.3. Prolonged Treatment with PM Causes Selective Enrichment of Microbial Communities

Data presented in Figure 1 and Figure 2 suggested, in addition to some age dependent changes, progressive changes in the gut microbiota due to PM treatment. Since progressive changes were noticeable with samples collected on day 30 and later, additional comparisons of microbial community profiles of control and PM-treated groups at these time points were made. As observed in heat maps of Figure 2C, relatively higher abundance of Verrucomicrobia and lower abundances of a major phylum Firmicutes and minor phyla were observed at days 30 and 45, but statistically significant differences were detected only with day 45 samples. At genus level, similar trends in the increase in *Bacteroides*, *Akkermansia*, *Luteolibacter*, and *Rubritalea* and decrease in *Blautia*, *Oscillospira*, and *Parabacteroides* were observed in PM recipient mice (Figure 2D).

Further compilation of OTUs at species level revealed a greater number of significant time dependent changes in the microbial communities of PM-treated mice compared to controls (Supplemental Figure S4). Further, differences in the abundances of higher numbers of communities over time (day 30 vs. day 45) were observed within PM-treated mice when directly compared to controls (Supplemental Figure S5). Notably, *Akkermansia muciniphila* showed significant increase within the gut microbiota in mice of both groups, with a more pronounced increased in PM-treated mice. Other communities that increased in the PM-treated mice at day 30 and day 45, compared to the pre-treatment time-point or control group at day 45 include an unclassified *Bacteroides* sp., a *Prevotella* sp., and *Bacteroides acidifaciens*. In PM-treated mice, there were significant decreases of greater than 5% in an unclassified member of the *Clostridiales* order at day 45 (*p* = 0.035) and an unclassified member of the family S24-7 of phylum Bacteroidetes at days 30 (*p* = 0.011) and 45 (*p* = 0.0081). Overall, these analyses show progressive changes/enrichment of microbial communities during PM treatment.

### 3.4. PM Treatment Affects Predictive Function of Gut Microbiota

**Figure 3:**
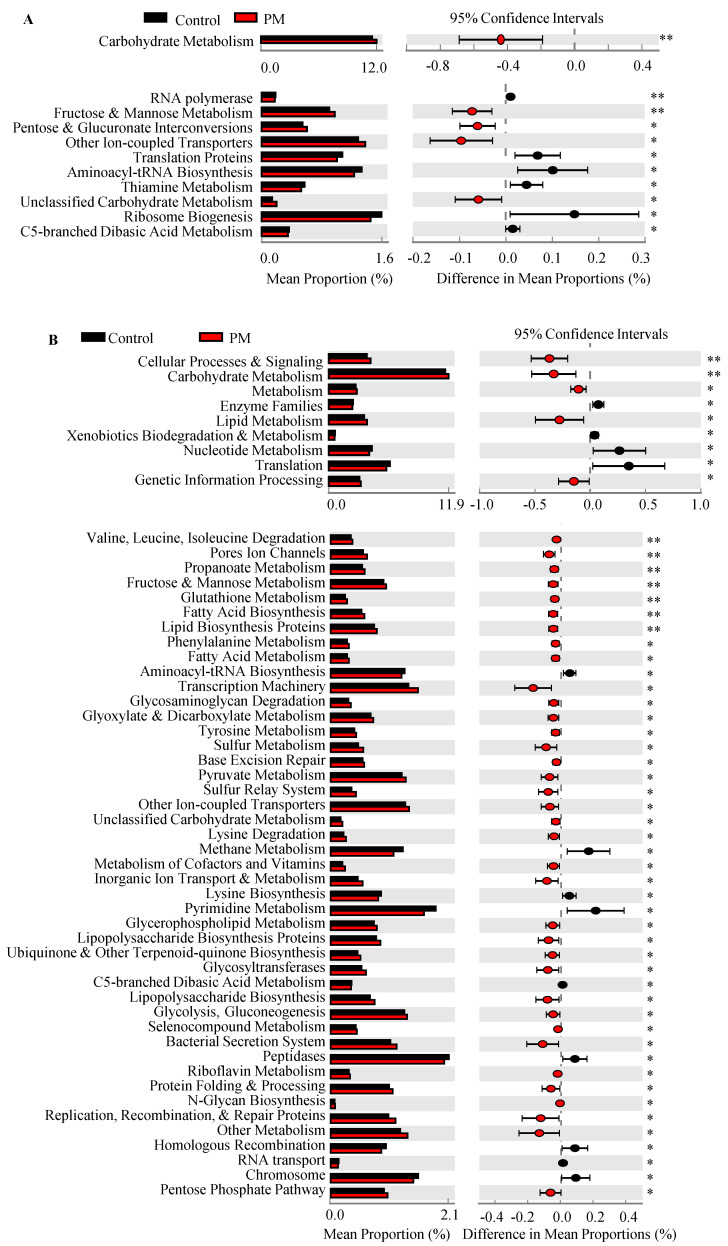
Predictive functional analysis of fecal microbiota in control and PM-treated mice. Predictive functional analysis of OTU data described in Figure 1 and Figure 2 was performed using PICRUSt and analyzed and visualized in STAMP showing designated functions at the 2nd (**A**) and 3rd (**B**) level of functional hierarchy at day 30 (upper panels) and 45 (lower panels) between control and PM-treated mice. FDR corrected *p*-values (Welch’s *t*-test) are shown. * *p* < 0.05 and ** *p* < 0.01.

Predicted functional profiles of fecal microbiota were generated from the OTU table using PICRUSt for control and PM-treated groups and analyzed for statistically significant differences. Regarding specific functions, no significant differences were observed in the functional levels in control mice between day 0 and 30 (data not shown). At both day 30 and 45, gut microbiota of the PM group showed a greater number of significantly different functions compared to control mice (Supplemental Figure S6). Comparisons between the different predictive functions of microbiota revealed significant overrepresentation of carbohydrate metabolism at both day 30 and 45 in PM-treated mice compared to controls (Figure 3A,B). Overall, OTUs of PM-treated mice showed significantly higher representation of predicted functions related to sugar processing and metabolism at both day 30 and 45, including fructose and mannose metabolism, glycosaminoglycan degradation, and pentose processing and utilization. As anticipated, microbiota of PM-treated mice at day 45 showed more altered functions compared to their day 30 counterparts. These included functions related to various carbohydrate processing and metabolism, fatty acid biosynthesis and metabolism, as well as certain amino acid metabolisms. Functions significantly downregulated at day 30 and/or day 45 include aminoacyl-tRNA biosynthesis, C5-branched dibasic acid metabolism, translation proteins, and methane metabolism. Representative predictive functions that were significantly overrepresented in PM-treated mice were analyzed further using the Local Mapper tool on iVikodak to determine which taxa were contributing the most to these particular functions. As shown in Supplemental Figures S7–S9, functions such as fructose and mannose metabolism, glycosaminoglycan degradation, and fatty acid biosynthesis, which are upregulated in PM-treated mice, are linked primarily to the *Bacteroides* genus. In PM recipient mice, there is a reduction in each of the examined functions linked to Firmicutes taxa compared to controls. Overall, these analyses, along with the microbial community profile data, show an association between PM treatment and progressive changes in the structure and function of gut microbiota.

### 3.5. Prolonged Treatment with PM Alters Intestinal Cytokine Profile

**Figure 4:**
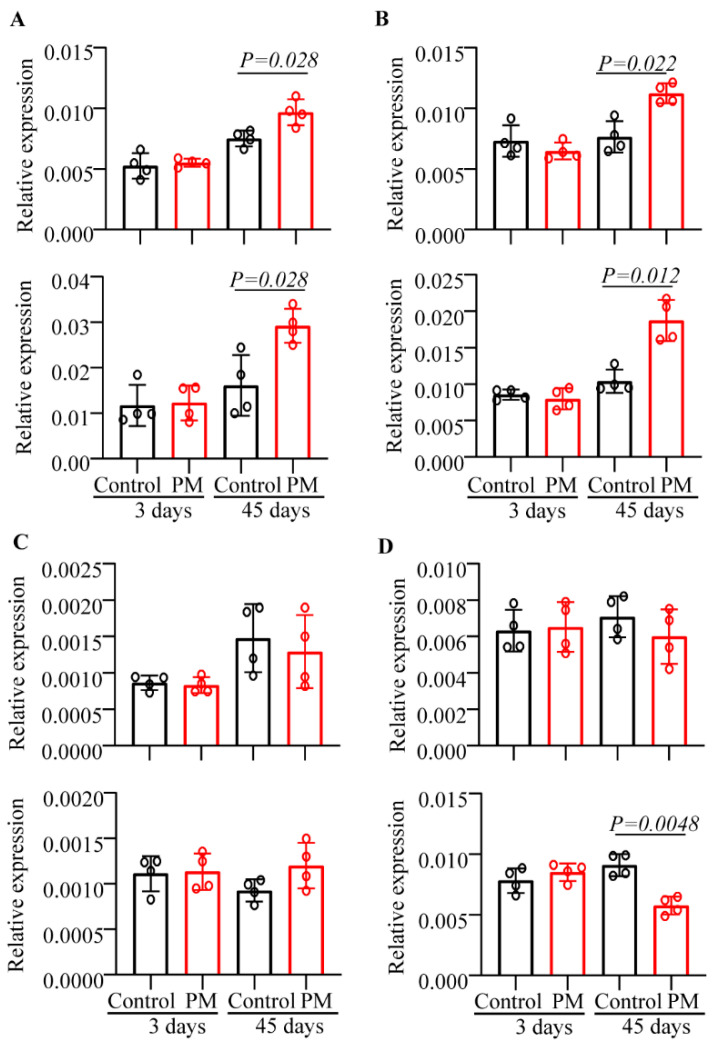
Prolonged treatment with PM causes immune activation in the gut mucosa. B6 mice were given PM by oral gavage for 3 or 45 consecutive days and euthanized after 24 h to harvest the intestine. cDNA preparations of the small intestine (SI; distal ileum; upper panels) and large intestine (LI; lower panels) were subjected to qPCR and the expression levels of cytokines and non-cytokine factors TNFα (**A**), IL10 (**B**), *Raldh1A2* (**C**), and IL6 (**D**) were compared. Expression levels relative to β-actin expression were plotted. *n*= 4 mice/group and the assay was performed in triplicate for each sample. *p*-values by Mann–Whitney test.

We reported that oral administration of YBG results in significantly higher expression of a large number of cytokines including IL10, TNFα, IL6, and IL1β as well as the tolerogenic factor Raldh1A2 in the small intestinal mucosa [5]. To determine if PM treatment has a similar impact on gut immune phenotype, cohorts of mice were treated for short-term (3 days) or long-term (45 days) as described above and the expression levels of various factors in the small and large intestines were determined by qPCR assay. As observed in Figure 4, mice that received PM for 3 days showed no detectable difference in the expression levels of *Il10*, *Tnfα*, *Il6* or *Raldh1a2* compared to their control counterparts. However, intestines, particularly large intestine, of mice that received PM for 45 consecutive days expressed significantly higher levels of *Il10* and *Tnfα* and lower levels of *Il6* compared to those of control mice. These observations, as indicated by differences in the cytokine expression levels mainly in the colon of mice that received prolonged treatment with PM, suggest that the PM-induced effect may primarily be altered microbiota, but not direct interaction with gut mucosa, dependent.

### 3.6. Prolonged Pretreatment with PM Diminishes Susceptibility to DSS Induced Colitis

**Figure 5:**
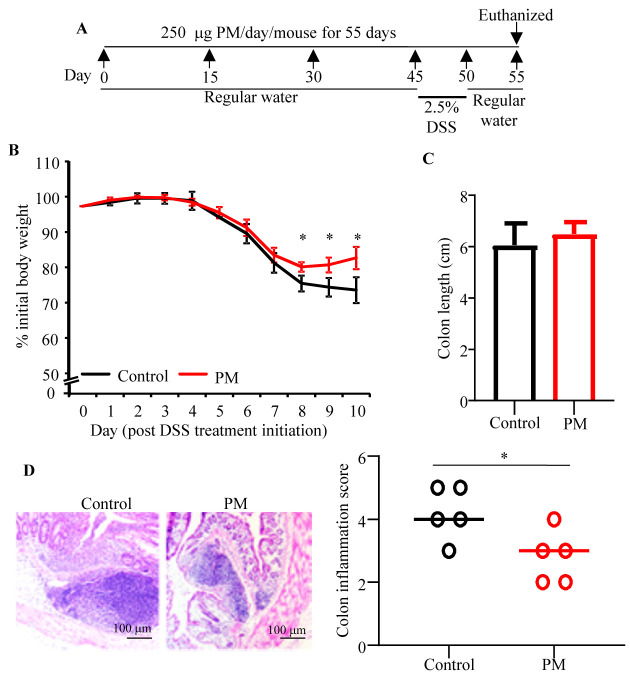
Impact of pre-treatment with PM on susceptibility to DSS-induced colitis. B6 mice were treated as depicted in panel (**A**). Bodyweights of individual mice were measured starting pre-DSS treatment (day 45) every day and mean ± SD values of changes in total body weights, relative to initial body weight (*n* = 5/group) are shown (**B**). *p*-value by unpaired *t*-test for each time-points. Mice were euthanized on day 55, colons were harvested, and their length was measured and mean ± SD values of length of colons from 5 mice/group are shown (**C**). H&E stained distal colon sections were evaluated for the degree of inflammation and representative images of sections (left panel) and mean ± SD values of inflammation severity scores of 5 mice/group (right panel) are shown (**D**). *p*-values by Fishers’ exact test comparing number of mice with grade ≤3 and ≥4 inflammation scores. * *p* < 0.05.

Our recent report showed that YBG, a structurally distinct β-glucan, can suppress colitis susceptibility when the mice were pre-treated [5]. To determine if PM treatment and associated structural and functional changes in gut microbiota impact colitis susceptibility, B6 mice were given PM for 55 consecutive days and DSS during the days 45–50, as shown in Figure 5A, and monitored for colitis associated loss of body weight. In addition, colon length and colonic inflammation were determined after euthanasia on day 55. As observed in Figure 5B, PM-treated mice were relatively less susceptible to weight loss compared to control mice. Significant differences in the body weight between control and PM-treated groups were observed starting day 8 post-DSS treatment initiation. Although colon lengths of control and PM-treated mice, upon euthanasia, were comparable (Figure 5C), overall histological injury scores in DSS-treated mice that received PM, compared to the control group, were significantly lower (*p* = 0.029) (Figure 5D). Overall, these observations show that pre-treatment with PM for a considerable duration can diminish susceptibility to colitis.

### 3.7. PM-Treated and Control Mice with Colitis Showed Distinct Immune Characteristics

**Figure 6:**
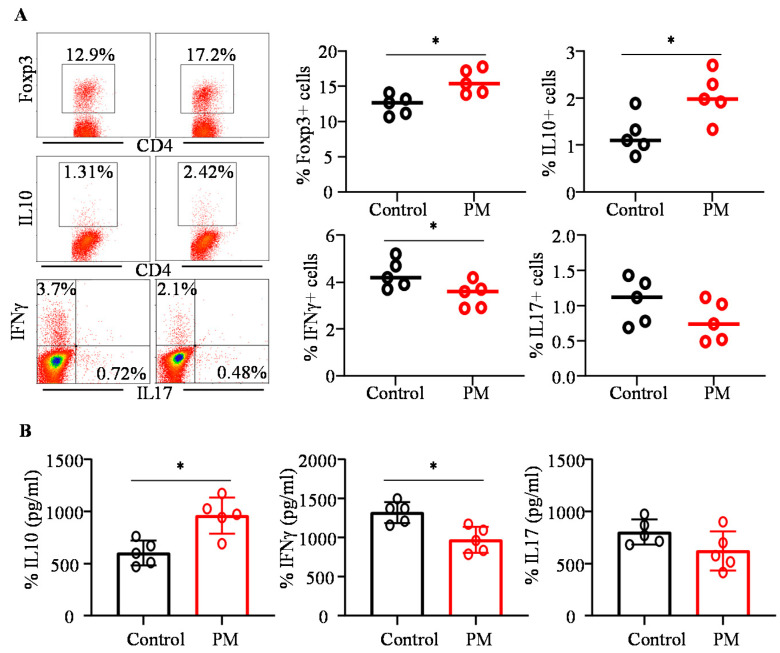
Impact of pre-treatment with PM on immune cells. B6 mice were treated as depicted in Figure 5A. (**A**) MLN cells were stained for Foxp3 and subjected to FACS analysis. Cells were also activated using PMA/Ionomycin in the presence of Brefeldin A for 4 h and subjected to intracellular staining for cytokines and analyzed by FACS. CD4+ population was gated for determining cell frequencies. Representative FACS plots (left panel) and mean ± SD frequencies of cells positive for indicated specific factors, 5 mice/group tested individually in duplicate, (right panel) are shown. (**B**) Immune cells were enriched from single cell suspension of colon tissue and cultured in the presence of anti-CD3 antibody for 24h and the spent media were subjected to Luminex technology-based multiplex assay. Mean ± SD values of concentrations of indicated cytokines produced by cells from 5 mice/group tested individually are shown. *p*-values by Mann–Whitney test. * *p* < 0.05.

Since PM fed mice showed less severe colitis compared to controls, immune cells from their gut associated tissues were examined for the phenotypic properties to realize if PM treatment related events caused immune modulation. As observed in Figure 6A, Foxp3+ T cell frequencies were profoundly higher in the MLN of PM fed mice compared to control mice (*p* = 0.018). Importantly, PMA/ionomycin stimulation ex vivo, followed by FACS analyses, showed that IL10+ T cell frequencies were significantly higher (*p* = 0.0226) and IFNγ+ T cell frequencies were lower (*p* = 0.0449) in PM fed mice. These observations were validated by multiplex cytokine assay that showed similar differences in the levels of IL10 and IFNγ secreted by colonic immune cells from PM-treated and control mice (*p* = 0.0119 and *p* = 0.0224 respectively), in response to ex vivo activation using anti-CD3 antibody (Figure 6B). Moreover, albeit not statistically significant, IL17 response was also relatively lower in PM-treated mice compared to controls. These results suggest that long-term PM treatment modulates the immune cell phenotype in gut mucosa.

## 4. Discussion

Previous work has shown β-glucans to be important modulators of the host immune response [3,4,5,16,17,33]. While our studies have shown profound effects of YBG on both the host’s gut microbiota as well as autoimmune disease/colitis outcomes [4,5], impact of another widely used β-glucan dietary supplement, PM of microalgae origin, on gut microbiota and immune function is largely unknown. The current study shows a significant prebiotic effect of PM upon prolonged oral administration on both shaping the gut microbiota and reducing susceptibility to gut inflammation. These changes include the altering of abundance of key bacterial taxa, influencing the community structure of gut microbiota, increasing the predicted levels of specific microbial functions related to carbohydrate metabolism, and improving disease outcomes with colitis through reduced inflammation and increased production of Tregs and anti-inflammatory cytokines.

Prolonged oral administration of PM appeared to cause progressive changes in the structure of gut microbiota. At the phylum level, PM-treated mice had significantly higher levels of Verrucomicrobia and significantly lower levels of Firmicutes. Previous studies using YBG have similarly found Verrucomicrobia to significantly increase and *Firmicutes* levels to profoundly decrease [4,5]. In contrast to YBG treatment, however, we did not observe significant changes in Bacteroidetes upon PM treatment. Differences in β-glucan structure may contribute to this observed difference, as the differing physical structure and properties of β-glucan can potentially alter the number of gut microbiota that preferentially utilize them [24]. While the *Bacteroides* genus profoundly increased upon PM treatment, the lack of noticeable effect on Bacteroidetes in PM-treated mice seems to be primarily due to significantly less abundance in the S24-7 family, a common member in the mammalian gut capable of metabolizing a diverse array of polysaccharides that seems to illicit a response by the innate immune system [34]. It appears that S24-7 family members do not have the ability to degrade PM and use it as an energy source for their growth and the reduction in their abundance may also be contributing to diminished susceptibility of PM recipient mice to gut inflammation.

The significant increase in Verrucomicrobia, particularly *Akkermansia muciniphila*, upon treatment with different types of β-glucans indicates an potential enrichment of bacteria in the mucosal lining of the intestines [35], which suggests either a prominent role of these bacteria in the metabolism of these complex carbohydrates or that β-glucan treatment contributes to increased mucus production in the gut that enriches this organism. Importantly, treatment with PM appeared to significantly increase the abundance of *Bacteroides* compared to controls. Several known species, including *B. acidifaciens*, *B. ovatus*, and *B. uniformis*, as well as unclassified *Bacteroides* spp. were found to be significantly more prevalent in PM-treated mice. *Bacteroides* spp. in the gut are typically associated with the production of SCFA like butyrate and acetate, which have important roles in the digestive and immune system function [36]. Therefore, decreased susceptibility to colitis in PM-treated mice may be linked to the increased levels of these bacteria. PM treatment also appears to downregulate the levels of certain bacteria within the gut, including *Oscillospira* spp., similar to YBG [4,5]. This genus has been reported to be a prominent member of the gut microbiome and appears to be linked with dietary habits and lower body fat in humans [37]. These bacteria are suggested to be unable to effectively metabolize complex fiber and tend to decrease upon higher supplementation of a plant-based diet [37], which could explain the observed decrease in their levels upon treatment.

The richness and diversity (α diversity) of the gut microbiota decreased significantly over time in both control and PM-treated mice, especially between days 15 and 45. It is possible that this effect may be related to microbiota maturation as well as aging. While gradual gut microbiota maturation during younger ages could diminish the diversity, it has been shown that aging can influence the structure of the gut microbial community [38,39]. Nevertheless, the more pronounced decrease in the richness and diversity of gut microbiota in PM-treated mice compared to control mice, suggests a prebiotic role for PM in further shaping the gut microbial community in addition to microbiota maturation and age-related changes. The degradation of this CDP appears to enrich certain bacteria significantly, reducing the diversity within the mouse gut. Similar to YBG treatment [5], PM treatment induced a decrease in richness of at least 150 estimated species within the gut microbiota. β diversity analysis showed significantly different community structures in control and PM-treated mice at day 30 and 45, indicating that β-glucan treatment causes a shift in the community composition and structure within the gut microbiota. Tighter clustering observed in PM-treated mice is indicative of the prebiotic effects of this CDP, which selectively enrich for potentially beneficial bacteria which contributes to diminished susceptibility to gut inflammation. Previous reports [40,41,42] indicate that a dysbiosis and a decrease in diversity of the gut microbiota, perhaps due to the environment, diet, antibiotics and genetics, is associated with the ulcerative colitis inflammatory phenotype. However, our results show a decrease in diversity along with a reduction in inflammation, indicating that the selective enrichment of certain, potentially host beneficial, taxa contributes to improved disease outcomes, despite the reduction in diversity. For example, gut colonization by specific microbial communities with the ability to promote immune regulation resulted in, irrespective of the gut microbial diversity, diminished susceptibility to gut inflammation. Hence, we believe that reduction in gut microbiota diversity due to selective enrichment of host beneficial community by prebiotics can favor enhanced immune regulation and suppressed colitis susceptibility [43,44,45].

In agreement with the structural changes in the gut microbiota, PM treatment showed a significant effect on its predictive functions, evidenced by significant overrepresentation of functions related to carbohydrate metabolism, fatty acid biosynthesis, and glycan biosynthesis and metabolism. This is consistent with utilization of CDPs and subsequent SCFA production [15,18,46]. Most of the overrepresented predicted functions observed in the gut microbiota of control mice were linked to Firmicutes, primarily to the genus *Proteiniborus*. Conversely, most of the overrepresented metabolic pathways observed in the gut microbiota of PM-treated mice were linked to *Bacteroides*, which has many species capable of SCFA synthesis and modulation of the immune system [5,36,47].

Importantly, PM treatment-associated shaping of the composition of the gut microbiota appears to impact the immune system, leading to suppression of susceptibility to DSS-induced colitis. Importantly, unlike YBG treatment [4], short-term treatment with PM appears to have no major impact on gut immune function. On the other hand, prolonged treatment did alter the gut immune phenotype, suggesting that this effect could be linked to altered gut microbiota. Our data showing a higher retention of body weight and a markedly lower inflammation in the colon of mice pretreated with PM, in addition to the significant increase in Treg frequencies and the anti-inflammatory cytokine IL10, indicate that PM treatment associated changes in gut microbiota structure and function are immune regulatory and host beneficial. In conclusion, the oral administration of PM as a dietary supplement is effective in altering the gut microbiota in composition, community structure and potentially the overall function, and in diminishing colitis susceptibility. The evidence presented herein suggests the prebiotic nature of PM oral treatment, which appears to significantly alter the function of the gut microbiota toward enhanced carbohydrate metabolism and contributes to reduced susceptibility to gut inflammation upon DSS-induced colitis. The decreased level of IFNγ and increased anti-inflammatory response upon PM treatment lend support toward this notion. Nevertheless, additional studies using gnotobiotic and microbiota depleted mice are needed in the future to fully understand the importance of specific microbial communities in diminishing susceptibility to gut inflammation upon dietary treatment using PM. Furthermore, as described in our recent report [5], BG consumption can increase the production of host beneficial microbial metabolites such as SCFAs which can modulate the host immune function. Hence, our future studies will also examine fecal and plasma metabolite profiles, particularly of microbial origin, to understand the molecular mechanism by which PM and PM-shaped microbiota enhance immune regulation and suppress susceptibility to colitis. Importantly, this study used highly purified PM to determine the true prebiotic value of this CDP. However, most microalgae BG preparations available as dietary supplements in the market are partially purified, and it is not known if they possess similar prebiotic properties. Furthermore, since this study was conducted in SPF mice under a well-controlled environment, clinical translational value of our observations needs to be validated through additional extensive pre-clinical studies using larger number and strains of animals and through human trials.

## Supplementary Materials

The following are available online at https://www.mdpi.com/2072-6643/12/8/2193/s1, Figure S1: Impact of treatment with PM on bodyweight. B6 mice were treated with saline (control) or PM for 45 days as described for Fig. 1, Figure S2: α and β diversity of the fecal microbiota in control and PMtreated mice, Figure S3: Gut microbiota compositional profiles of control and PM-treated Mice, Figure S4: Taxonomic profile showing significant time dependent changes at the species level within control (A) and PM treated (B) mice, analyzed using STAMP, Figure S5: Relative abundances of microbial communities at the species level were determined for fecal samples collected from control and PM treated mice of day 30 (A) and day 45 (B) employing STAMP, Figure S6: Differences in the prevalence of specific functions at the 2nd levels of functional hierarchy were examined in control mice between days 0 and 45 (A) and PM treated mice between days 0 and 30 (B) and days 0 and 45 (C) employing STAMP, Figure S7: Taxa contributing toward the function of fructose and mannose metabolism at day 45 in control and PM treated mice, analyzed employing the Local Mapper tool in iVikodak, Figure S8: Taxa contributing toward the function of glycosaminoglycan degradation at day 45 in control and PM treated mice, analyzed employing the Local Mapper tool in iVikodak, Figure S9: Taxa contributing toward the function of fatty acid biosynthesis at day 45 in control and PM treated mice, analyzed using the Local Mapper tool in iVikodak.

## Abbreviations

BG	β-glucan
CDP	complex dietary polysaccharides
DSS	dextran sulfate sodium
FACS	Fluorescence-activated cell sorting
H&E	hematoxylin and eosin
KEGG	Kyoto encyclopedia of genes and genomes
LDL	low-density lipoprotein
MLN	Mesenteric lymph nodes
OUT	operational taxonomic units
PICRUSt	Phylogenetic Investigation of Communities by Reconstruction of Unobserved States
PM	paramylon
PMA	phorbol myristate acetate
SCFA	short-chain fatty acid
SPF	specific pathogen-free
STAMP	Statistical Analysis of Metagenomic Profiles
T1D	type 1 diabetes
YBG	yeast β-glucan
