# Supplementary material for: Dynamics of Structural and Functional Changes in Gut Microbiota during Treatment with a Microalgal β-Glucan, Paramylon and the Impact on Gut Inflammation
Source: Nutrients. 2020 Jul 23;12(8):2193. doi: 10.3390/nu12082193 (PMC7468787; doi:10.3390/nu12082193)
Supplement: Supplementary file 1 [file nutrients-12-02193-s001.pdf]

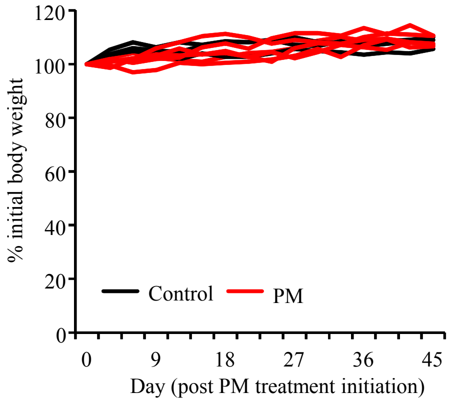

Supplemental Figure 1. Impact of treatment with PM on bodyweight. B6 mice were treated with saline (control) or PM for 45 days as described for Fig. 1. Bodyweights of individual mice were measured starting pre-treatment (day 0) every third day and changes in total body weights, relative to initial body weight (n=4 for control and 5 for PM group) are shown.

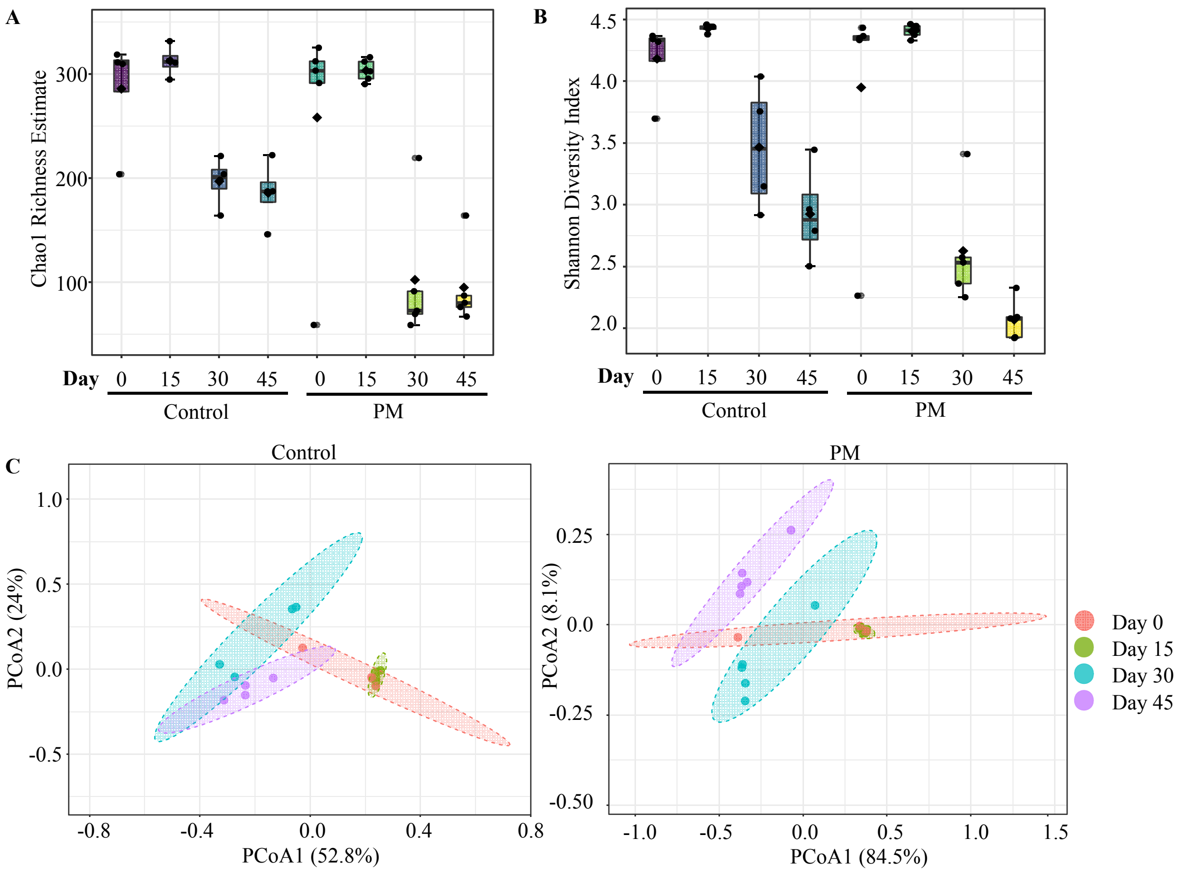

Supplemental Figure 2. α and β diversity of the fecal microbiota in control and PM-treated mice. α and β diversity analyses were performed using 16S rRNA gene sequencing data of fecal samples from control (n=4) and PM treated (n=5) mice collected before (day 0) and at various time-points (days 15, 30 and 45) during the treatment. Chao1 estimates (A) and Shannon indices (B) were employed to evaluate richness and diversity within each sample, respectively. Statistical significance was assessed using t-tests with FDR correction value of 0.05. Panel C shows principal coordinate analysis (PCoA) examining the community structure differences between samples over time for each treatment. This analysis was done using Bray-Curtis dissimilarity approach and the statistical significance was evaluated using PERMANOVA and mentioned in the text.

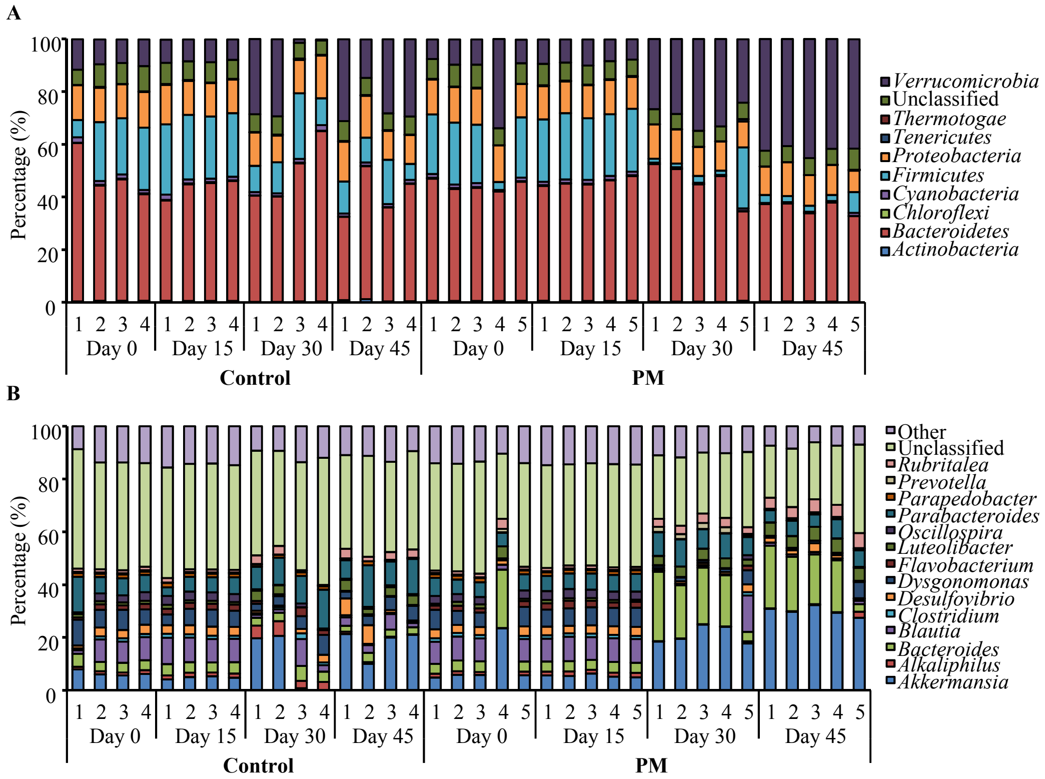

Supplemental Figure 3. Gut microbiota compositional profiles of control and PM-treated mice. Composition of microbial communities within each individual mouse on each sampling date are shown at the phylum (A) and genus (B) level.

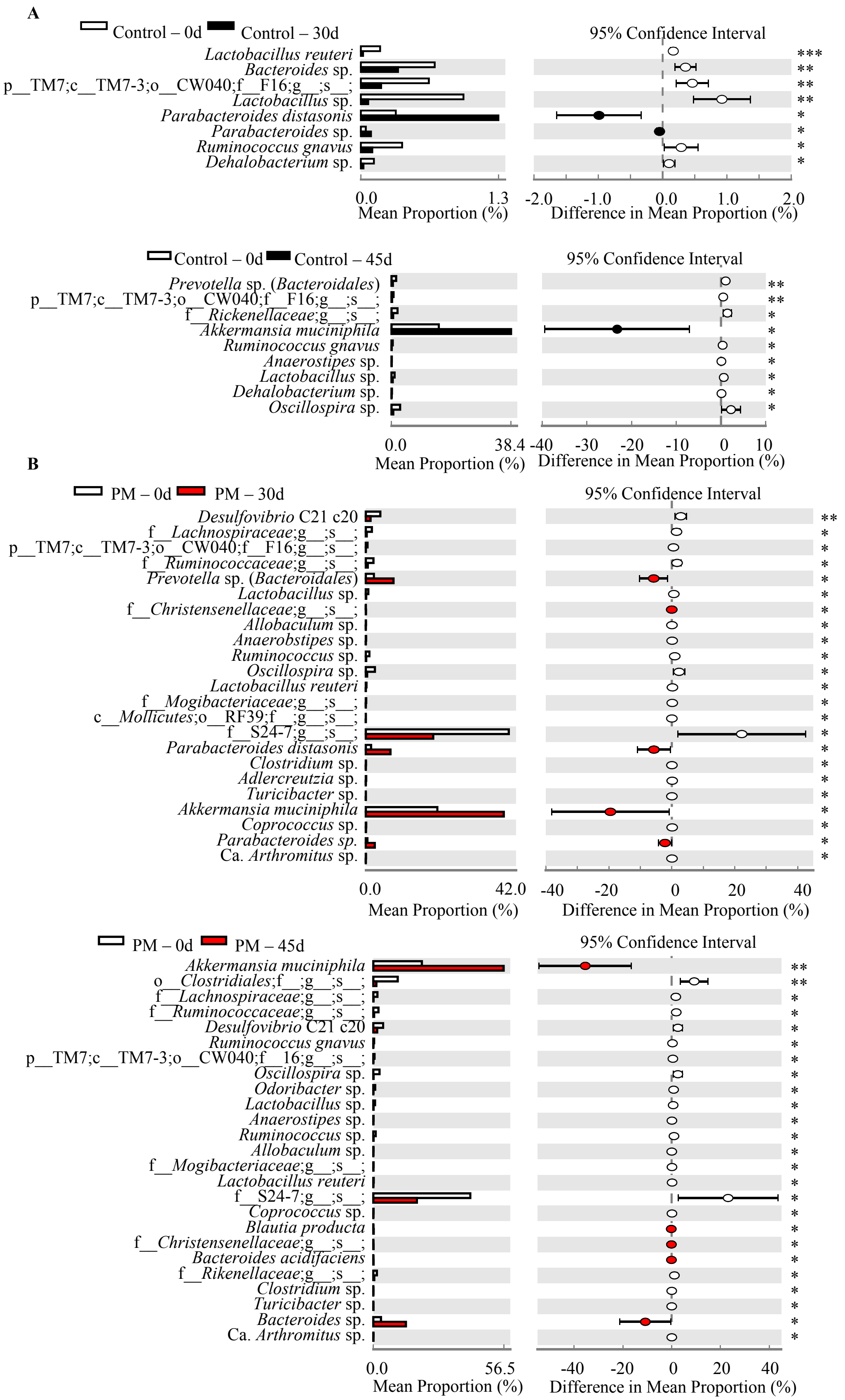

Supplemental Figure 4. Taxonomic profile showing significant time dependent changes at the species level within control (A) and PM treated (B) mice, analyzed using STAMP. *$p<0.05$, **$p<0.01$ and ***$p<0.001$.

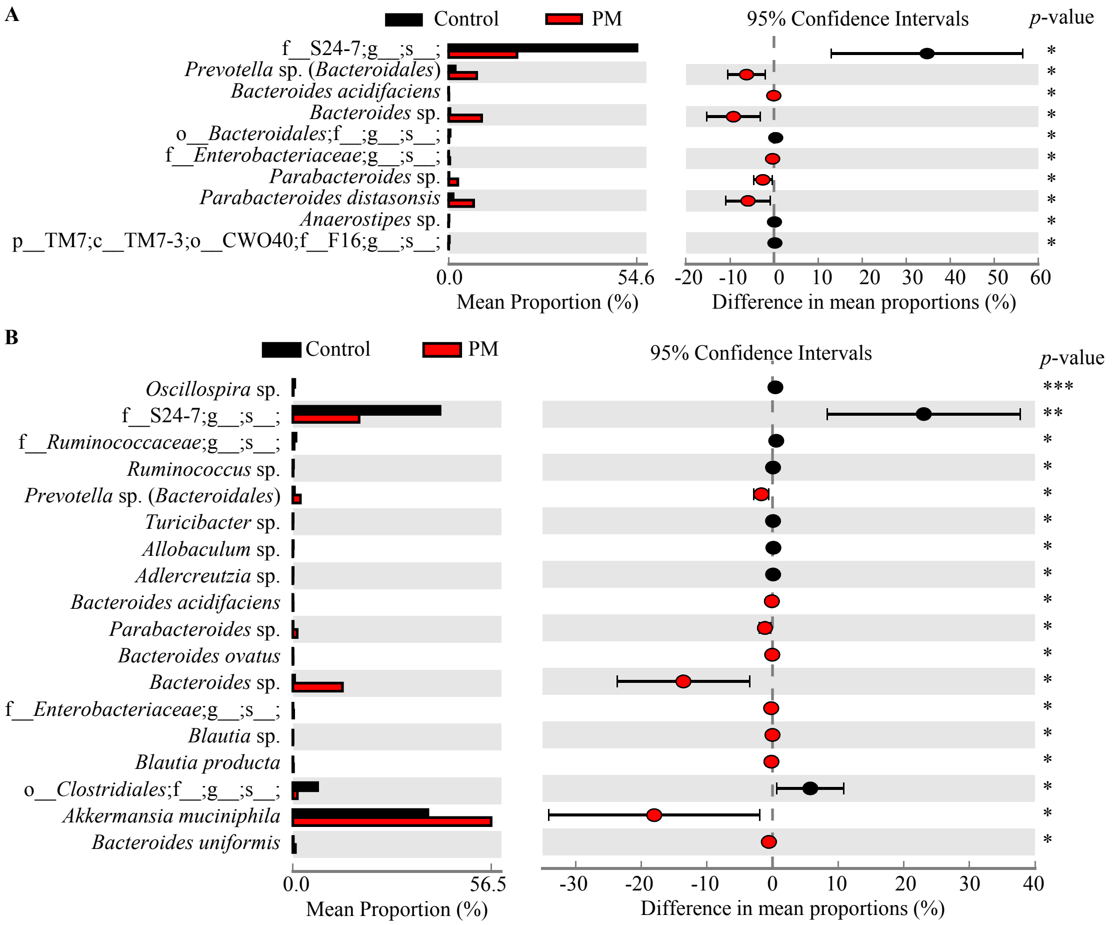

Supplemental Figure 5. Relative abundances of microbial communities at the species level were determined for fecal samples collected from control and PM treated mice of day 30 (A) and day 45 (B) employing STAMP. FDR corrected p-values (Welch's t-test) are shown. $^{*}p<0.05$, $^{**}p<0.01$ and $^{***}p<0.001$.

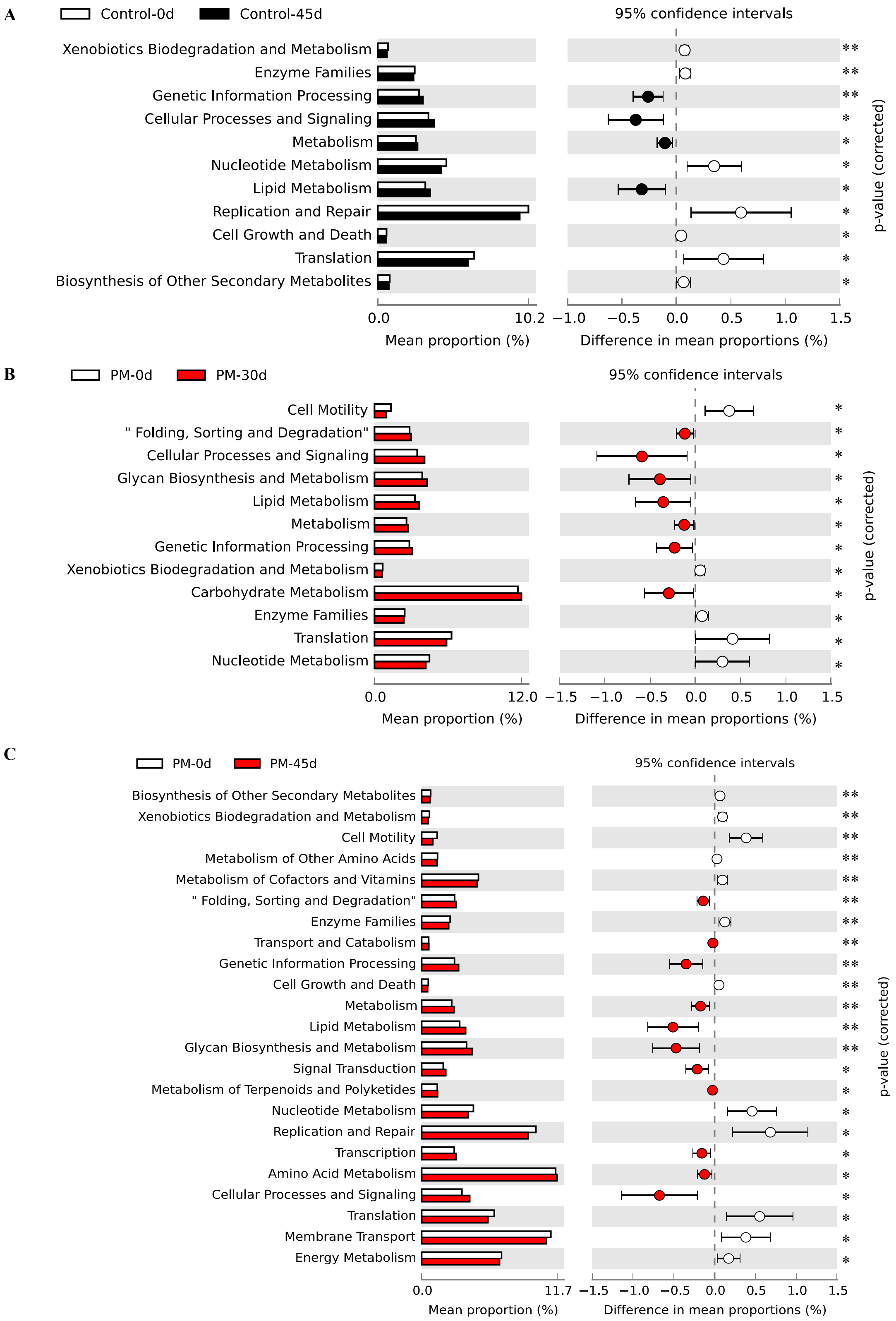

Supplemental Figure 6. Differences in the prevalence of specific functions at the 2nd levels of functional hierarchy were examined in control mice between days 0 and 45 (A) and PM treated mice between days 0 and 30 (B) and days 0 and 45 (C) employing STAMP. *p<0.05 and **p<0.01.

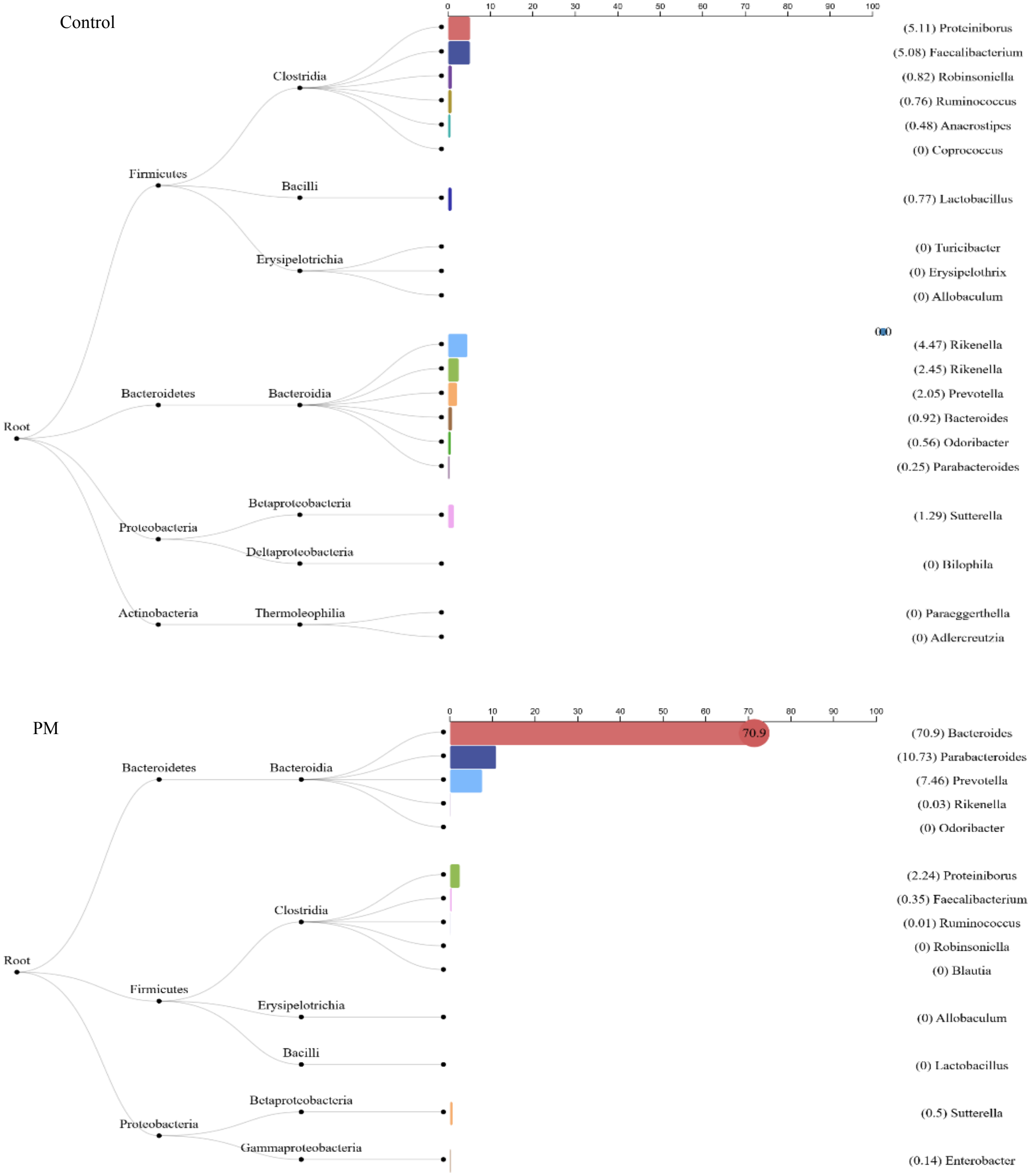

Supplemental Figure 7. Taxa contributing toward the function of fructose and mannose metabolism at day 45 in control and PM treated mice, analyzed employing the Local Mapper tool in iVikodak.

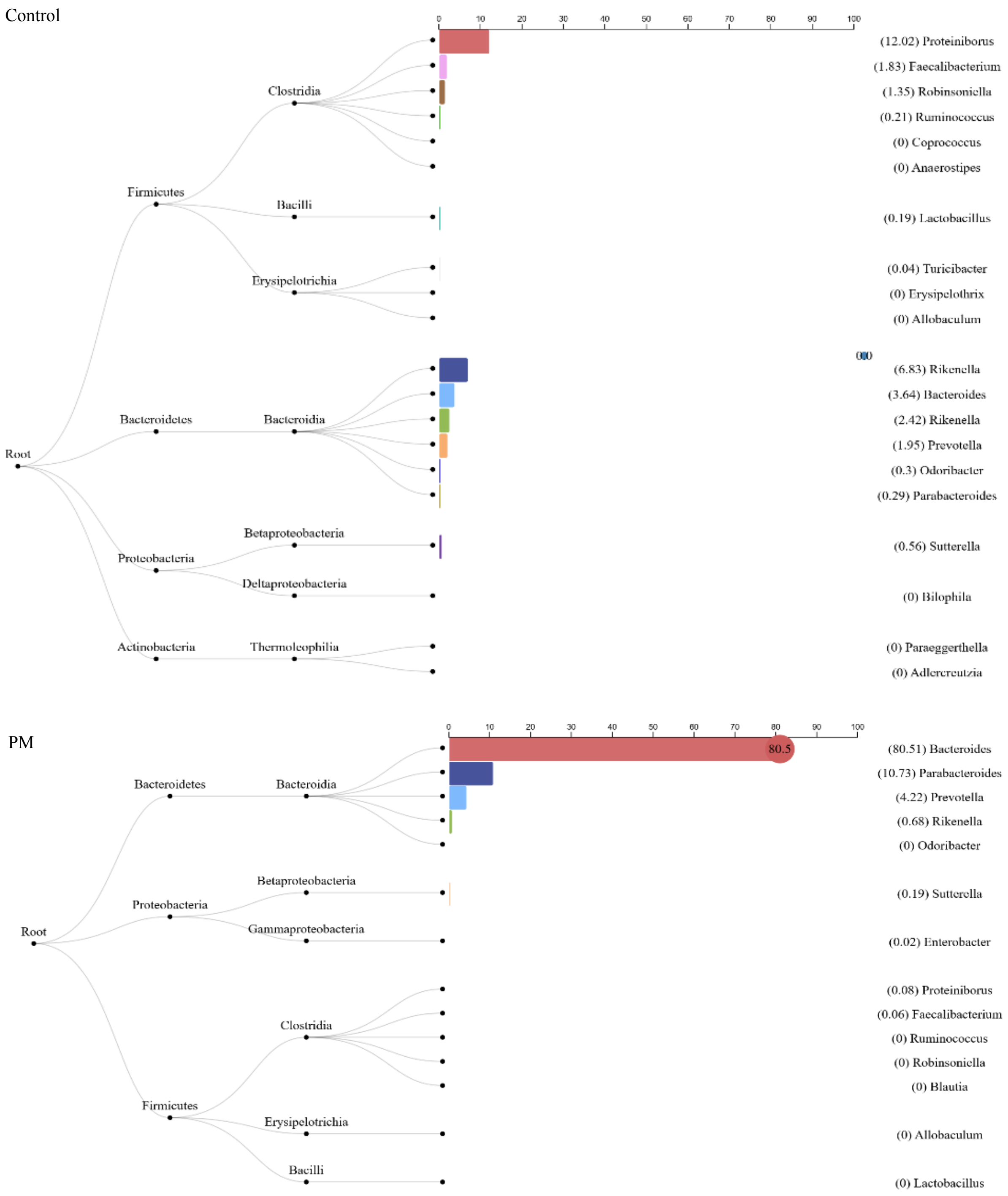

Supplemental Figure 8. Taxa contributing toward the function of glycosaminoglycan degradation at day 45 in control and PM treated mice, analyzed employing the Local Mapper tool in iVikodak.

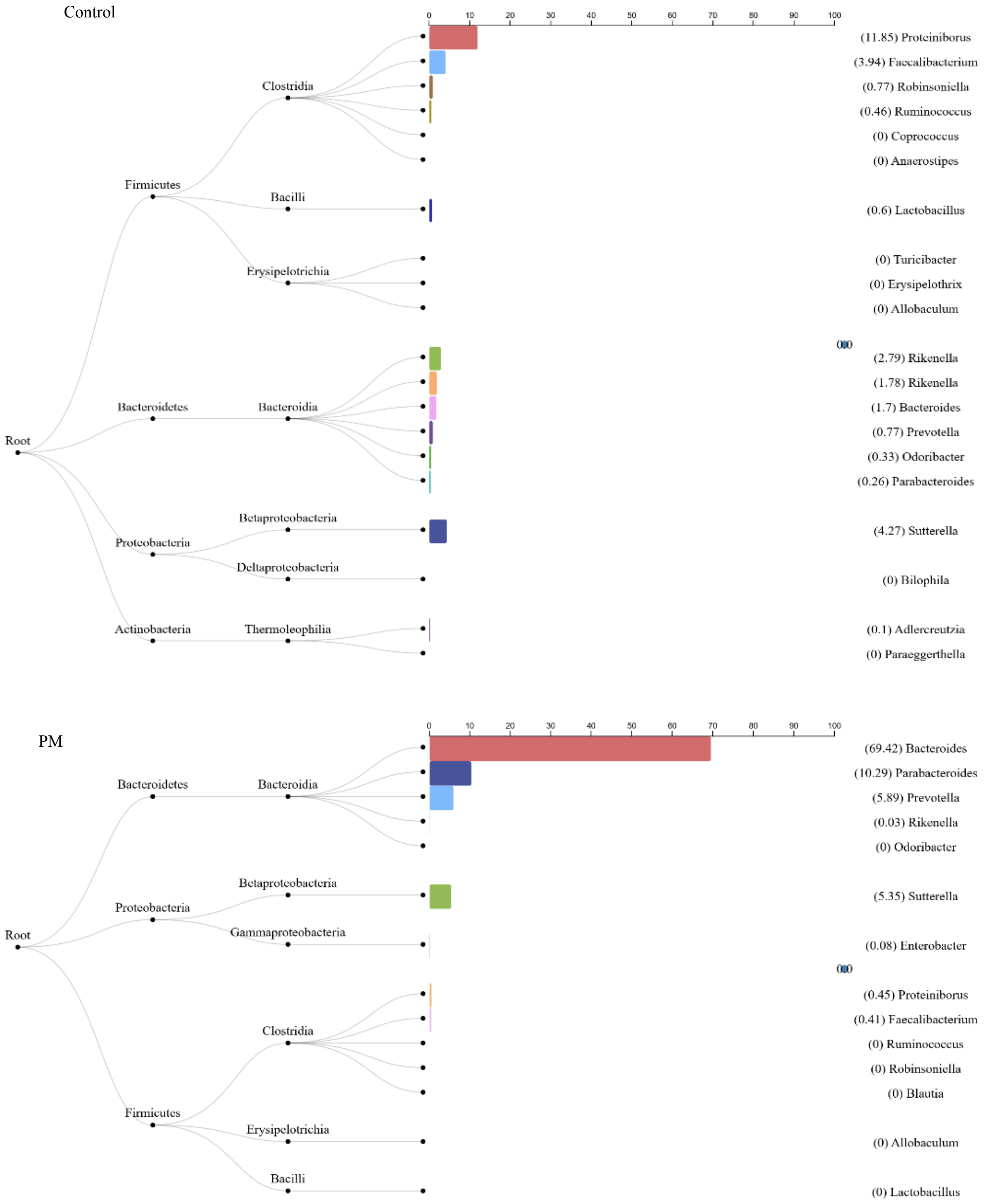

Supplemental Figure 9. Taxa contributing toward the function of fatty acid biosynthesis at day 45 in control and PM treated mice, analyzed using the Local Mapper tool in iVikodak.
